# Maternal High-Fat Diet Alters the Characteristics of Astrocytes and Worsens the Outcome of Stroke in Rat Offspring, Which Improves After FGF21 Administration

**DOI:** 10.3389/fcell.2021.731698

**Published:** 2022-01-13

**Authors:** Yanxuan Li, Mengqi Lin, Ping Lin, Nengzhi Xia, Xiaokun Li, Li Lin, Yunjun Yang

**Affiliations:** ^1^ Department of Radiology, The First Affiliated Hospital of Wenzhou Medical University, Wenzhou, China; ^2^ School of Pharmaceutical Sciences, Wenzhou Medical University, Wenzhou, China

**Keywords:** FGF21 (fibroblast growth factor 21), DOHaD (development origins of health and disease), stroke, astrocyte, maternal high fat diet

## Abstract

**Background:** Maternal high-fat diet (MHFD) has been shown to increase susceptibility to neurological disease in later offspring, but the underlying mechanism is not clear. Fibroblast growth factor 21 (FGF21) has been reported to have a neuroprotective effect in stroke, but its mechanism of action remains unknown. In this study, we investigated the mechanism of the effect of MHFD on stroke in offspring in adulthood and the mechanism by which FGF21 acts on stroke and restores neurological function.

**Methods:** We performed transcriptome sequencing analysis on D21 neonatal rats. Bodyweight and blood indicators were recorded in the adult rats after MHFD. FGF21 was administered 7 h after photochemical modeling twice a day for three consecutive days.

**Results:** We found numerous mRNA changes between the MHFD group and a normal maternal normal diet (MND) group at D21, including genes related to astrocyte and PI3K/Akt pathways. The body weight, blood glucose, and triglycerides of the MHFD offspring were higher, ischemic lesions were larger, the number of activated astrocytes was lower, and the neurological function score was worse than that of the MND group. After FGF21 administration, WB and qPCR analyses showed that astrocytes and the PI3K/Akt pathway were upregulated, while NF-κB and inflammatory cytokines expression were inhibited in stroke and peri-stroke regions.

**Conclusion:** Taken together, we conclude that MHFD alters the characteristics of astrocytes and other transcriptome changes in their offspring, leading to a worse prognosis of stroke, while FGF21 plays a neuroprotective role by inhibiting NF-κB and inflammatory factors and activating the PI3K/Akt pathway and activating more astrocytes in the MND group than the MHFD group.

## Introduction

In early life, exposure to environmental contaminants may affect the metabolism of the central nervous and endocrine systems, leading to inflammation and apoptosis (Omran, 1971). The Developmental Origins of Health and Disease (DOHaD) concept articulates a relationship between metabolism in adult life and early life events, such as pregnancy, lactation, and adolescence (Suzuki, 2018). There is growing evidence that maternal high-fat diet (MHFD) can cause health problems in adult offspring, such as an increased susceptibility to ischemic stroke, which is a leading cause of death and disability (Bejot et al., 2007). Lin et al. (2016), Lin et al. (2018) found that MHFD could greatly affect adult cerebrovascular health by regulating central brain-derived neurotrophic factor expression and HPA axis, as well as through ET-1 manner in remodeling of both structure and function. Although these articles explain some of it, the underlying mechanism is still unclear. Astrocytes are important innate immuno-regulators in the brain. They control the brain’s vascular input during development and are involved in various neurological disorders. Studies have shown that astrocytes play important roles during the early, middle, and late stages of stroke (Ullian et al., 2001; Christopherson et al., 2005; Koehler et al., 2009; Eroglu and Barres, 2010; Allen et al., 2012; Zhou et al., 2019). And astrocytes are highly sensitive to environmental changes, and thus can be easily influenced (Pekny et al., 2019). However, the changes of astrocyte characteristics in stroke after MHFD remain unclear. In addition to astrocytes, inflammation also plays important roles in stroke. In the inflammatory response, NF-κB is considered as a typical pro-inflammatory signaling pathway, mainly based on the activation of nuclear factor kappa-B (NF-κB) by pro-inflammatory cytokines such as interleukin-1 (IL-1) and tumor necrosis factor-alpha (TNF-α) (Barnes, 1997; Karin and Ben-Neriah, 2000; Karin et al., 2006; Kaltschmidt and Kaltschmidt, 2009; Dresselhaus and Meffert, 2019; Howell and Bidwell, 2020). The conventional treatment for acute ischemic stroke is recombinant tissue plasminogen activator (rtPA), which restores ischemic cerebral blood flow (Rabinstein, 2017). However, because of its narrow therapeutic window and the effects of other variables, there is an urgent need to explore other neuroprotective drugs (Prabhakaran et al., 2015). Fibroblast growth factor 21 (FGF21) regulates blood glucose and lipid metabolism. A study (Wang et al., 2018) has shown that blood levels of FGF21 increased in high-fat feeding mice compared to normal feeding mice. Also, FGF21 is a hormone that acts on receptors in the nervous system, regulating sympathetic nervous system activity, metabolism, and body weight. Importantly, FGF21 does not have mitotic activity, which ensure its safety for clinical application. In addition, FGF21 can cross the blood-brain barrier, which makes it a potential treatment for central nervous system diseases (Staiger et al., 2017; Li, 2019, 21). Also, studies have shown that FGF21 can play a neuroprotective role in stroke by enhancing the blood-brain barrier, microglia regulation and inhibiting inflammation (Jiang et al., 2020; Wang et al., 2020). However, the interaction between astrocytes and FGF21 has not been reported.

Based on the above, we investigated the effects of MHFD on astrocytes in the brain and the likelihood of stroke in adulthood and the effects of FGF21 administration.

## Materials and Methods

### Reagents and Antibodies

The primary antibodies used for immunofluorescence included anti-p-NF-κB (No. 3033) and anti-GFAP (No. 3670) purchased from Cell Signaling Technology (Danvers, MA, United States) and ProteinTech (Wuhan, China). The secondary antibodies were Donkey anti-rabbit IgG H&L (Alexa Fluor 647) and Donkey anti-mouse IgG H&L (Alexa Fluor 488) purchased from AbCAM (Cambridge, MA, United States).

Primary antibodies for Western blotting were anti-NF-κB (No. 8248), anti-p-NF-κB (No. 3033), anti-PI3K (No. 20584-1-AP), anti-p-PI3K (No. 4228T), anti-GFAP (No. 16825), and anti-β-actin (No. 660009) purchased from Cell Signaling Technology (Danvers, MA, United States), Abcam (Cambridge, MA, United States), and ProteinTech (Wuhan, China). The secondary antibodies were donkey anti-rabbit IgG H&L (HRP) (ab150075) purchased from Abcam (Cambridge, MA, United States).

The corresponding reagents and kits used in this study included TRIzol reagent (Qiagen, Duesseldorf, Germany), IQTM SYBR Green Supermix (Bio-Rad, Hercules, CA, United States), PrimeScript RT reagent kits (Takara, Shiga, Japan), QuantiTect rt-PCR kit (Qiagen, Duesseldorf, Germany), Mirneasy Micro Kit (Qiagen, Duesseldorf, United States), and TaqMan gene expression analysis kit (ThermoFisher Scientific, Fremont, CA, United States).

FGF21 was supplied by key Laboratory of Biopharmaceutical, School of Pharmaceutical Sciences, Wenzhou Medical University. FGF21 was extracted and purified from Escherichia coli according to Wang et al. (2010).

### Animal Preparation

Female and male Sprague-Dawley rats (7 weeks old) were purchased from the cLaboratory Animal Center of the Chinese Academy of Sciences (Shanghai, China) and raised at Wenzhou Medical University under the following conditions: 22°C, unlimited access to food and water, 12/12 h light/dark exposure (lighting was started at 7 am). All surgical and animal experiments were approved by the Animal Protection and Use Committee of Wenzhou Medical University.

**FIGURE 1:**
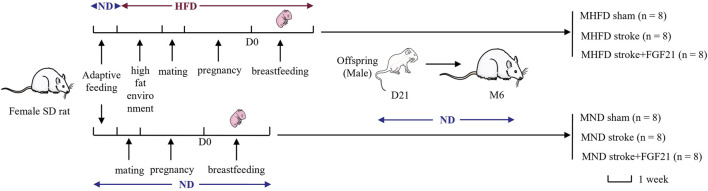
Experimental procedures and flow diagram of this study.

During the first week of adaptive feeding, all rats were fed a normal diet (5.3% fat, corn oil, 57.4% carbohydrate, 21.2% protein, 4.6% fiber; 360 k calories/100 g of food; Medicience Ltd., Jiangsu, China). After 1 week, half of the female SD rats were randomly selected to be fed a high-fat diet (25.7% fat, 19.5% protein, 41.3% carbohydrate, 3.5% fiber; estimated fats: stearic acid 1.99%, palmitic acid 4.5%, palmitoleic acid 0.12%, linoleic acid 2.58%, oleic acid 6.86%, arachidonic acid 0.19%, a-linolenic acid 0.25%; 470 k calories/100 g of food; Medicience Ltd.). After 7 days, they were allowed to mate, become pregnant, and suckle their young (all of which were fed a high-fat diet). The other half of the female rats continued to be fed a normal diet and were allowed to mate, become pregnant, and suckle their young after 21 days. The first day of birth of offspring SD rats was defined as D1. D21 was the last day of lactation, and all the male offspring of SD rats in the following two groups {MHFD and maternal normal diet (MND) groups} were fed with normal feed until the 6th month of photothrombotic stroke modeling. All animals were randomly divided into MND sham group, MND stroke group, MND stroke + FGF21 group, MHFD sham group, MHFD stroke group and MHFD stroke + FGF21 group (flow diagram can be seen in Figure 1).

### Photothrombotic Stroke Procedure

Photothrombotic stroke was induced as follows: SD rats were anesthetized by isoflurane, and the scalp was cut to expose the skull. Ten minutes before light exposure, rats were intraperitoneally injected with 15 mg/kg Bengal rose (Sigma-Aldrich, St. Louis, MO, United States), and then fixed on a stereotaxic device, and the end of a 4 mm diameter optical fiber cable was placed on the top of the skull at the location where embolization was determined. After 10 min of Bengal rose injection, a cold light source and a green bandpass filter (KL1600 LCD, SCHOTT, Zeiss, Germany) were turned on to illuminate the exposed skull. When the area had been lit for 15 min, the light was turned off, and the incision was sutured and disinfected. Sham rats underwent the same procedure but did not receive Bengal rose injection. The rats in MND stroke + FGF21 and MHFD stroke + FGF21 groups were intraperitoneally injected with FGF21 twice a day at a dose of 1.5 mg/kg for 3 consecutive days at 6 h after modeling.

### Magnetic Resonance Imaging

Cerebral infarct size was evaluated using 3-T clinical magnetic resonance imaging with a 72 mm linear transmitter coil and an animal surface receiver coil for rat brain imaging. The rats were anesthetized by an intraperitoneal injection of 10% chloral hydrate. During the MRI scan (10 min per rat), the rats were placed on a blanket to maintain a normal body temperature (36–37°C). We obtained broad MRI acquisitions as follows: 1) T1-weighted images (repetition time [TR]/echo time [TE] = 2005/15 ms) and 2) T2-weighted images (TR/TE = 4,900/120 ms). Image analysis was performed using ImageJ software (National Institutes of Health).

### Behavior Assessment

Behavioral tests (tensile and balance beam tests) were conducted on days 1, 2, and 3 after ischemic injury. The same investigators carried out the evaluation procedure, while being blinded to the experimental groups to minimize discrepancies in the experiment. The mice were euthanized after behavioral tests were completed.

### Immunofluorescence Staining

After euthanasia, rats were perfused with normal saline through the left ventricle, and the whole brain tissue was removed, fixed with 4% paraformaldehyde, dehydrated, and waxed. The whole-brain wax block was placed on a micrograph to prepare the brain sections. Sections (5 μm thick) were sealed at room temperature for 1 h in 5% goat or donkey serum. Next, the tissue sections were co-incubated with anti-GFAP and anti-NF-κB antibodies at 4°C overnight and then incubated with appropriate antibodies. Finally, the cells were stained with DAPI (Beyotime, Shanghai, China). Images were obtained using a fluorescence microscope (Leica, Japan).

### Quantitative Real-Time Polymerase Chain Reaction

Total RNA from infarct and peripheral brain tissue was extracted using QIAGEN’s (Cat.74004) RNEasy Micro kit. RNA concentrations were quantified using a NanoDrop spectrophotometer (Thermo Fisher Scientific, MA, United States). Then, 1 μg of total RNA was used to synthesize cDNA by using iScript reverse Supermix for RT-qPCR (RR037A, TaKaRa, Japan). Next, SYBR-based real-time PCR (Bio-Rad, Hercules, CA, United States) was used to detect the total transcription of IL-1β, TNF-α, and IL-6. The oligonucleotide PCR primers listed in Table 1 were purchased from Sango Biotech (Shanghai, China).

**TABLE 1:** Information of primers.

Primer	Sequence	
IL-6	F	ctc​cca​aca​gac​ctg​tct​ata​c
	R	cca​ttg​cac​aac​tct​ttt​ctc​a
TNF-α	F	atg​tct​cag​cct​ctt​ctc​att​c
	R	gct​tgt​cac​tcg​aat​ttt​gag​a
IL-1β	F	aag​cct​cgt​gct​gtc​gga​cc
	R	tga​ggc​cca​agg​cca​cag​gt
18s	F	gcc​atg​cat​gtc​taa​gta​cgc
	R	ccg​tcg​gca​tgt​att​agc​tc

F forward primer, R reverse primer.

### Western Blotting

Total proteins from stroke and peri-stroke brain tissues were extracted using a protein extraction reagent containing 1% protease and phosphatase inhibitors. First, the concentration of protein was determined using the absorbance method. Next, the same amount of protein (60 μg) was isolated on an SDS-PAGE gel and then transferred to a PVDF membrane. After sealing with whole milk, primary antibodies (anti-GFAP, anti-NF-κB, anti-p-NF-κB, anti-PI3K, anti-p-PI3K, anti-*β*-actin diluted 1:200) were used and incubated overnight under a shaking table at 4°C. On the second day, the combined primary antibody bands were washed three times with Tris-buffered saline–Tween 20 and incubated at room temperature in 1:10,000 diluted secondary antibodies for 1 h. Finally, immunoreactive protein bands were prepared using an enhanced chemiluminescence (ECL) kit, and the band density was quantified using Image Lab 3.0 software (Bio-Rad).

### Measurement of Blood Index

2 ml of blood is taken from offspring rats through the tail vein before anesthesia for modeling after 12 h of fasting. The blood samples were solidified at 4°C for 2 h and centrifuged at 3,000 rpm/min for 15 min. The serum samples were transferred to a new test tube and stored at −80°C. FGF21 concentration was measured by an ELISA kit (Boster, United States). The concentration of albumin, cholesterol, triglycerides and blood glucose were measured by an auto biochemical analyzer (IDEXX catalyst One, United States).

### Transcriptome Sequencing

At D21, three neonatal rats were randomly selected from both groups, and their brains were collected and frozen in a -80 refrigerator. Total RNA was extracted and used for transcriptome sequencing (LC-Bio Technologies (Hangzhou) Co., Ltd.).

### Statistical Analysis

Data are expressed as the mean ± SEM. Statistical differences between the multiple data sets or two groups were evaluated using one-way ANOVA and post-hoc analysis with Bonferroni correction was done to identify statistical differences between specific groups or unpaired t-tests in two groups in GraphPad Prism Edition 8 (GraphPad Software Inc., San Diego, CA, United States). Statistical significance was set at *p* < 0.05.

## Results

### Transcriptome Sequencing Analysis of the Brains of the Offspring of the MHFD and MND Groups on D21

**FIGURE 2:**
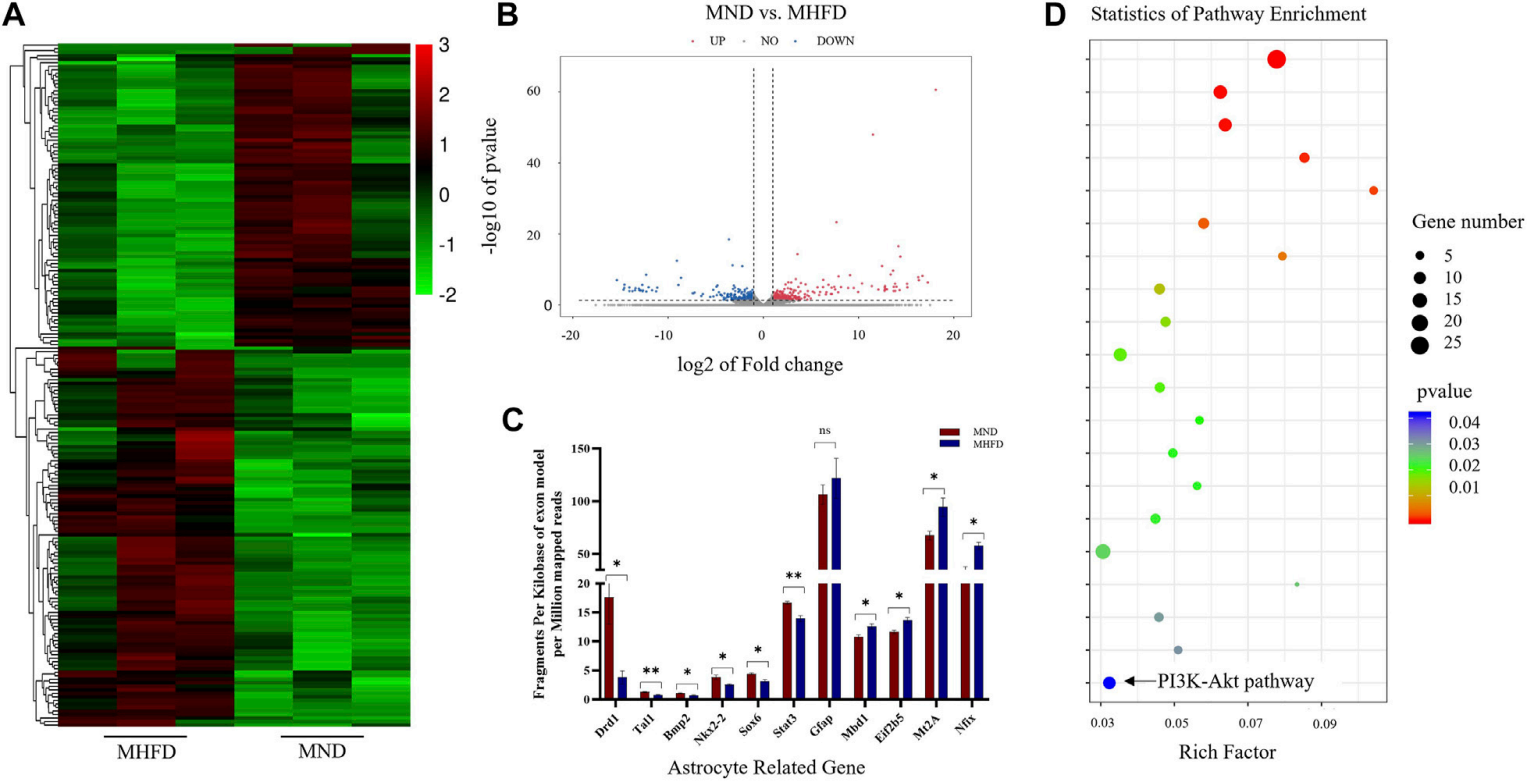
Comparison of brain transcriptome sequencing between MHFD and MND groups on D21. **(A)**. Heat maps of the top 200 different genes between the two groups. **(B)**. The volcano map of differentially expressed genes in the brain tissues of the two groups of neonatal rats on D21. The down-regulation and up-regulation are indicated by blue and red dots, respectively, with *n* = 3 for each group. **(C)**. 11 genes in KEGG analysis associated with astrocytes. **(D)**. KEGG analysis of RNA-seq data revealed the top 20 regulatory pathways that changed in both groups. *p*-values are shown in different colors, and bubble sizes indicate the number of genes in each pathway.

The researchers performed transcriptome sequencing analyses of the brains between the MHFD and MND groups (D21). Differences in gene expression were observed in the brains of the D21 MHFD group compared with the MND group (Figure 2A). Among the differentially expressed genes, 297 and 264 genes were downregulated and upregulated, respectively (Figure 2B). The transcriptomics analysis also showed that the MHFD offspring had 6 downregulated and 4 upregulated astrocyte-related genes, compared to MND (Figure 2C) among all genes. The proteins encoded by these altered genes are involved in astrocytes genesis (Gfap), differentiation (Bmp2, Nkx2-2, Sox6, Stat3, Nfix, Mbd1, and Eif2b5), activation (Mt2A), and fate commitment (Tal1), which were indicated by Gene Ontology (GO) database.

Representative differences in active pathways between the two groups were observed using the Kyoto Encyclopedia of Genes and Genomes (KEGG) enrichment analyses (Figure 2D). Of note, KEGG is a large-scale molecular data set generated through genome sequencing and other high-throughput experimental techniques that contribute to the understanding of advanced functions of biological systems. Notably, the PI3K-Akt signaling pathway plays a key role in cell survival and susceptibility. In addition, it also acts as a downstream signaling molecule after FGF21 acts on FGFR.

### Evaluation of Characteristics Between MHFD and MND Groups

To assess the effects of MHFD on offspring in rats, we measured and recorded the body weights of both MHFD and MND groups from the end of lactation (21 days) to 6 months and measured blood glucose, triglycerides cholesterol, and albumin on the day before inducing ischemia.

**FIGURE 3:**
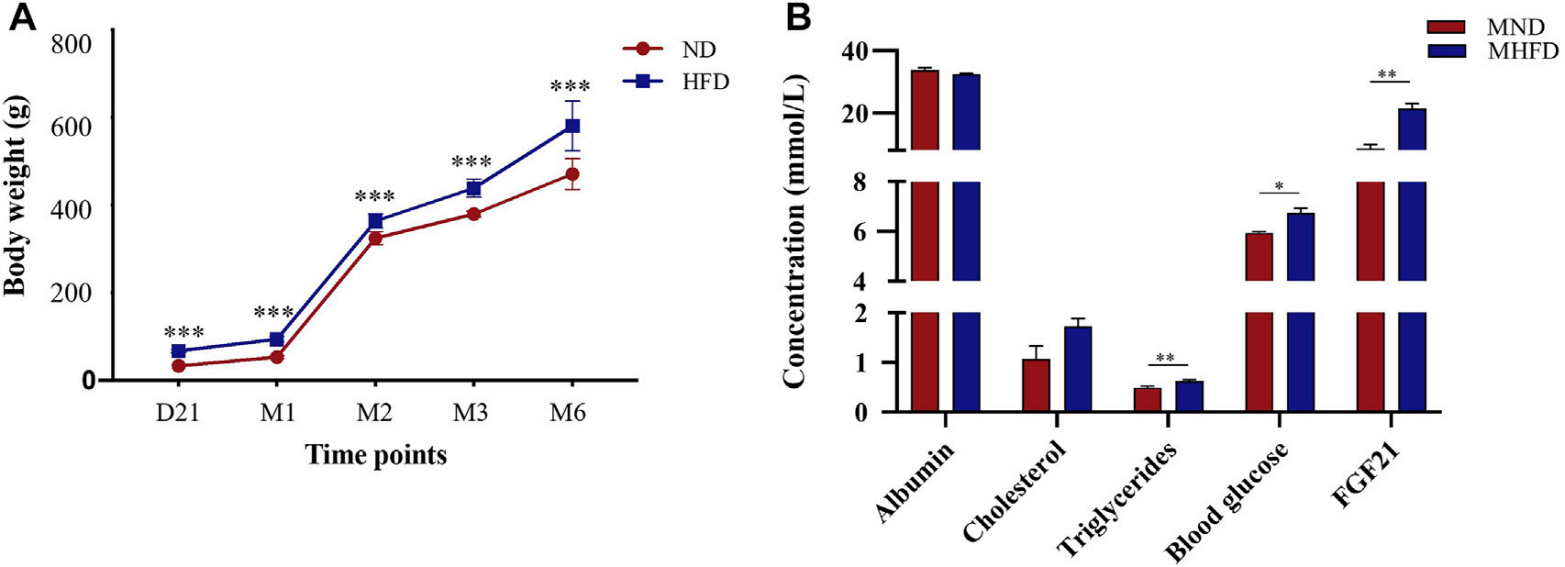
Evaluation of characteristics between MHFD and MND newborn/middle-aged SD rats. The comparisons between the two groups at various time points are shown in **(A)** (*n* = 8). ***p < 0.001, Unpaired *t*-test. The comparison of blood indexes of the two groups before modeling is shown in **(B)** (*n* = 4). *p < 0.05, **p < 0.01, Unpaired t-test.

As shown in Figure 3A, the MHFD and MND offspring bodyweights were different throughout D21 to M6. Blood sample analyses showed no differences in albumin or cholesterol levels between the two groups (Figure 3B). However, triglycerides (*p* < 0.05), blood glucose (*p* < 0.01) and FGF21 (*p* < 0.01) were higher in the blood of the MHFD group than in that of the MND group.

### Comparison of Cerebral Infarction and Neurological Deficit Between MHFD Stroke+/-FGF21 and MND Stroke+/-FGF21 Groups

We scanned the brains of the rats by using MRI and delineated the maximum infarct + edema area by using ImageJ to quantify the cerebral infarct size on Day 3. In addition, balance beam and tension test scores were used to assess neurological deficits.

**FIGURE 4:**
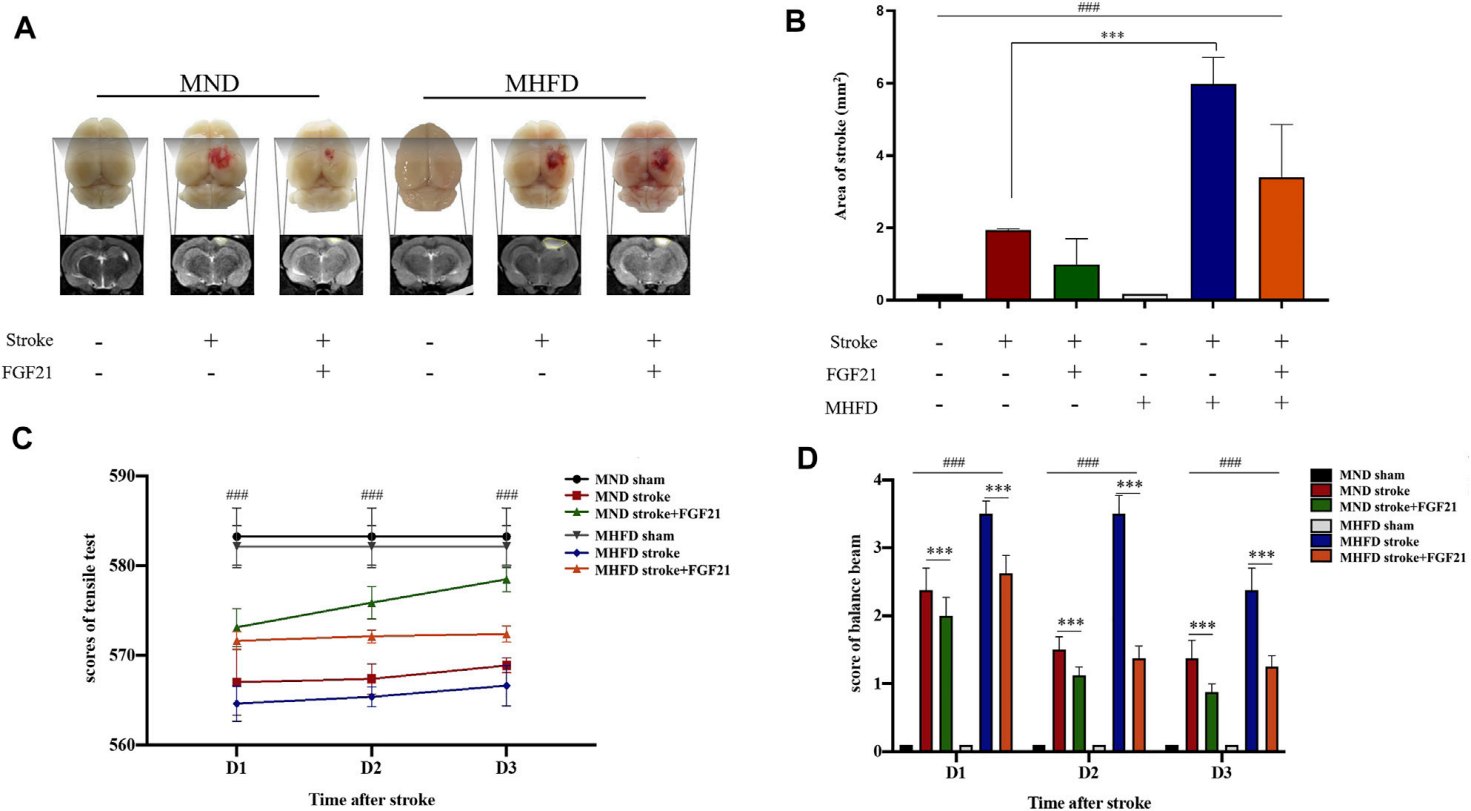
Comparison of the volume of cerebral infarction and neurological deficit between HFD stroke+/-FGF21 and HD stroke+/-FGF21 groups. **(A)**. Cerebral infarct size and MR images of the four groups. **(B)**. Evaluation of infarct size on magnetic resonance images (*n* = 3). ###p < 0.0001, one-way ANOVA; ***p < 0.0001, post-hoc analysis with Bonferroni correction. **(C)**. Grip strength tests were performed in each group at 1, 2, and 3 days after cerebral infarction (*n* = 8). ###*p* < 0.0001, one-way ANOVA. **(D)**. Balance beam score of each group at 1, 2 and 3 days after cerebral infarction (*n* = 8). ###p < 0.0001, one-way ANOVA; ***p < 0.0001, Unpaired *t*-test.

As shown in Figure 4, the infarct size of MHFD offspring was larger than that of the MND group. After FGF21 administration, the size of the cerebral infarction decreased in both groups.

After the injury, tensile tests showed that the MND group had greater tensile strength than the MHFD group, and tensile strength improved after rats were given FGF21, compared with the no treatment group. Balance scores revealed that the MND + FGF21 group had the best neurological recovery following ischemia, while the MHFD group had the worst neurological function after cerebral infarction, and the other two groups were in between. Behavioral data from days 1–3 and MRI data from Day 3 showed rats treated with FGF21 following ischemia had better neurological function recovery than untreated rats.

### FGF21 Plays a Neuroprotective Role in Ischemic Brain Tissue by Activating the Astrocyte and PI3K-Akt Signaling Pathways, Thereby Inhibiting Phospho-NF-κB

To study how FGF21 acts on the infarction’s size, we used immunofluorescence and WB analyses to study the changes in astrocytes and inflammatory cytokines (NF-κB) in the infarction and surrounding areas in different groups.

**FIGURE 5:**
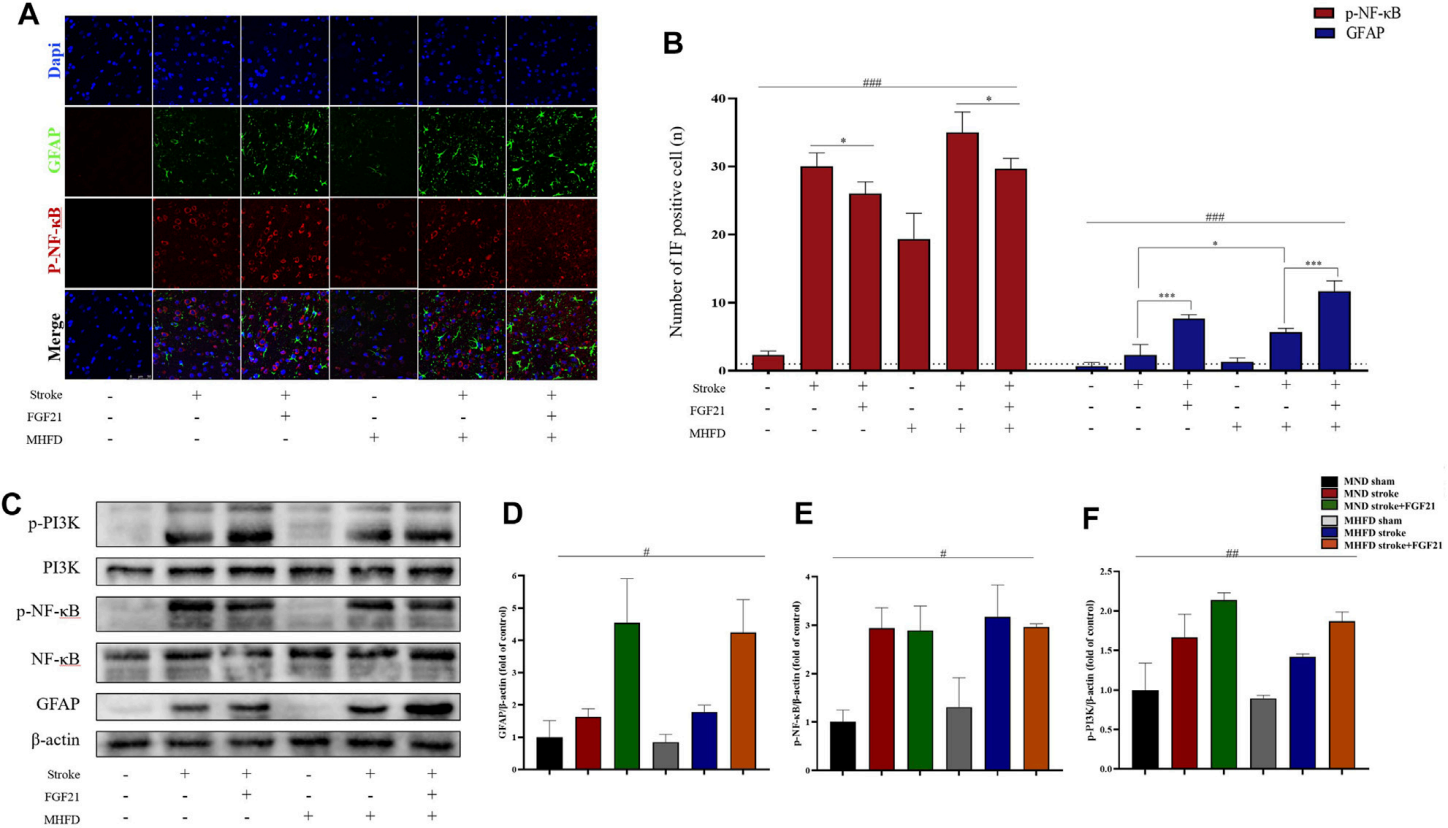
FGF21 plays a neuroprotective role by activating astrocytes and inhibiting NF-κB expression. **(A)**. Representative immunofluorescence images of GFAP and NF-κB. **(B)**. Quantification of immunofluorescence staining data showing the expression of GFAP positive and p- NF-κB positive cells. ###*p* < 0.0001, one-way ANOVA; *p < 0.05, ***p < 0.0001, post-hoc analysis with Bonferroni correction. **(C)**. Representative images of p-PI3K, PI3K, p-NF-κB, NF-κB, *β*-actin and GFAP expression in the stroke and peri-stroke brain tissues detected by western blot. **(D–F)**. Densitometric analysis for the protein expression of GFAP, p-PI3K and *p*-NF-κB. #*p* < 0.05, ##*p* < 0.01, one-way ANOVA.

As shown in Figure 5, the peri-stroke astrocytes of rats were activated after inducing ischemia, and there were more astrocytes in the MHFD group than in the MND group. After FGF21 administration, the number of astrocytes increased significantly in both groups.

### FGF21 Inhibits Inflammatory Response in the Area of Peri-stroke

**FIGURE 6:**
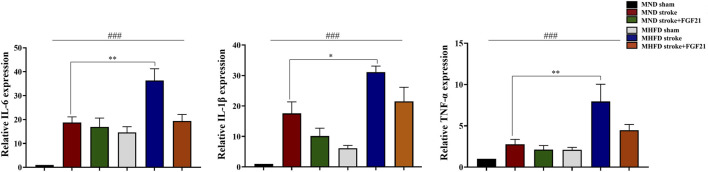
FGF21 inhibits inflammatory response in the stroke and peri-stroke brain tissues. ###*p* < 0.0001, one-way ANOVA; *p < 0.05, **p < 0.01, post-hoc analysis with Bonferroni correction.

We extracted brain tissue from the ischemic lesions and surrounding tissue to further examine the effect of FGF21 on inflammatory cytokine release. We evaluated gene expression by real-time PCR (Figure 6). The inflammation in MHFD was more severe than MND group.

FGF21 administration after cerebral infarction effectively reduced the production of inflammatory cytokines in stroke and peri-stroke regions in both groups.

## Discussion

The study of DOHaD investigates relationships between early life events and adult metabolism. The period between pregnancy, lactation, and puberty is a window of time in which any small event can shape a person’s metabolism for life (Almeida et al., 2019). Our transcriptome sequence analyses showed that expression of genes involved in activation, migration, and development of astrocytes in the MHFD-offspring was different from that in the MND offspring. These results suggest that MHFD leads to biogenetic and metabolic changes in astrocytes in the nervous system. It has been demonstrated that MHFD may be involved in the epigenetic regulation of gene expression (Suzuki, 2018; Bordeleau et al., 2020). For example, Bordeleau et al. (2020) showed how dietary habits during pregnancy and parenting, especially a fat-rich diet, affect offspring peripheral immune priming. Also, a study found that rat’s hippocampal neuron transcriptome was altered in MHFD offspring, affecting cognitive function (Sacks et al., 2018). Our transcriptome sequence analyses also showed changes in the PI3K-Akt pathway. The PI3K/Akt signaling pathway regulates cell survival, growth, proliferation, angiogenesis, transcription, translation, and metabolism (Katso et al., 2001; Hennessy et al., 2005). This result suggests that MHFD makes the brain more susceptible to the consequences of the disease, which also confirms the earlier findings that MHFD is associated with more severe ischemic damage.

In addition to the changes observed at D21, we also found differences in adult weight and blood markers of MHFD-offspring compared to those of MND offspring, consistent with previous studies. For example, a study by Ng et al. (2010) showed that long-term MHFD resulted in weight gain, *β*-cell dysfunction, and impaired glucose metabolism in offspring. In addition, Barker and others (Hales and Barker, 1992) found an epidemiological association between low birth weight and later glucose metabolic disorders, including type 2 diabetes. Moreover, consisting with previous studies that MHFD affects rat’s nervous system development, increasing offspring’s susceptibility to adverse stroke outcomes in adulthood. Lin et al. (2018) found that MHFD exposure renders adult offspring brains more susceptible to ischemic injury. In addition, the importance of early life challenges in modulating adult offspring’s susceptibility to brain injury has been demonstrated in animal models, such as momentary separation of mother and infant and neonatal immune challenges. Similarly, a mother’s high-fat diet makes the brain of the offspring more vulnerable to ischemic damage and other cerebrovascular diseases in adulthood. Our results also confirmed that offspring of MHFD are prone to more severe cerebrovascular accident injury than MND offspring.

Furthermore, our experiments showed that FGF21 is neuroprotective in ischemic stroke. When FGF21 was administered to rat brains following ischemic injury, infarction size reduced, and their performance in balance and tension strength assessments increased. Both *in vitro* and *in vivo* studies have demonstrated that FGF21 is neuroprotective. For example, Wang et al. (2020) found that FGF21 treatment promoted recovery from stroke. Jiang et al. (2020) found that rFGF21 protects against acute BBB leakage. Another study found that FGF21 protects against HFD-induced cognitive impairment, and that FGF21 may regulate the pathogenesis of diseases caused by MHFD (Cordner et al., 2019). FGF21 is neuroprotective in aging rat brains by reducing the formation of advanced glycation end products, improving behavioral performance, and alleviating d-galactose-induced oxidative stress (Yu et al., 2015).

When we explored the neuropharmacological mechanisms of FGF21, we found that infarcted and surrounding tissue had upregulated PI3K/Akt signaling pathway and GFAP expression. At the same time, the expression of NF-κB and other inflammatory cytokine was inhibited treated with FGF21. NF-κB is believed to be a major regulator of inflammation, and IL-1β, IL-6 and TNF-α are rapidly released in response to tissue injury or infection (Karin et al., 2006). Other studies also confirmed that FGF21 could inhibit inflammation and protects against neuronal death (Jiang et al., 2020; Wang et al., 2020). PI3K-Akt pathway is regulated by many growth factors and regulators (Hennessy et al., 2005). Although the biological outcome of FGF activation of downstream pathways depends on the cellular environment, it has been shown that PI3K/Akt mostly promotes cell survival (Beenken and Mohammadi, 2009; Goetz and Mohammadi, 2013). Thus, upregulation of the PI3K-Akt signaling pathway may be the decisive mechanism of increased stroke susceptibility in adult rats. Astrocytes account for 15–20% of all cells in the rodent brain and support neurotransmission by circulating neurotransmitters and stromal delivery of energy and nutrients (Sofroniew, 2009; Pekny et al., 2019). In addition, astrocytes regulate reactive glial hyperplasia accompanied by upregulation of many astrocyte genes, such as GFAP (Buffo et al., 2010; Pekny and Pekna, 2016). Within a few days of ischemic injury, astrocytes proliferate in the penumbra of stroke. Some migrate toward the infarction boundary, limiting the area of injury and preventing invading white blood cells from invading healthy brain tissue (Bush et al., 1999; Myer et al., 2006). We found ischemic injury activated astrocytes, which migrated to the infarct area. Additionally, FGF21 administration to ischemic lesions increased the number of activated astrocytes in rat brains.

## Conclusion

We conclude that an MHFD alters offspring’s astrocyte transcriptome and increases their susceptibility to cerebral infarction in adulthood. Additionally, FGF21 treatment effectively improves neurological function after stroke. The neuroprotective mechanism of FGF21 may be through the activation of astrocytes and inhibition of neuroinflammation. FGF21 may be developed into a new and powerful approach to treat ischemic stroke.

## Data Availability

The original contributions presented in the study are publicly available. The data presented in the study are deposited in the Gene Expression Omnibus repository, accession number GSE189679.

## References

[B1] Almeida D. L. Pavanello A. Saavedra L. P. Pereira T. S. de Castro-Prado M. A. A. de Freitas Mathias P. C. (2019). Environmental Monitoring and the Developmental Origins of Health and Disease. J. Dev. Orig Health Dis. 10, 608–615. 10.1017/S2040174419000151 31130151

[B41] Allen N. J. Bennett M. L. Foo L. C. Wang G. X. Chakraborty C. Smith S. J. (2012). Astrocyte Glypicans 4 and 6 Promote Formation of Excitatory Synapses via GluA1 AMPA Receptors. Nature 486, 410–414. 10.1038/nature11059 22722203 PMC3383085

[B2] Barnes P. J. (1997). Nuclear Factor-Κb. Int. J. Biochem. Cel Biol. 29, 867–870. 10.1016/s1357-2725(96)00159-8 9304801

[B3] Beenken A. Mohammadi M. (2009). The FGF Family: Biology, Pathophysiology and Therapy. Nat. Rev. Drug Discov. 8, 235–253. 10.1038/nrd2792 19247306 PMC3684054

[B4] Bejot Y. Benatru I. Rouaud O. Fromont A. Besancenot J. P. Moreau T. (2007). Epidemiology of Stroke in Europe: Geographic and Environmental Differences. J. Neurol. Sci. 262, 85–88. 10.1016/j.jns.2007.06.025 17761197

[B5] Bordeleau M. Lacabanne C. Fernández de Cossío L. Vernoux N. Savage J. C. González-Ibáñez F. (2020). Microglial and Peripheral Immune Priming Is Partially Sexually Dimorphic in Adolescent Mouse Offspring Exposed to Maternal High-Fat Diet. J. Neuroinflammation 17, 264. 10.1186/s12974-020-01914-1 32891154 PMC7487673

[B6] Buffo A. Rolando C. Ceruti S. (2010). Astrocytes in the Damaged Brain: Molecular and Cellular Insights into Their Reactive Response and Healing Potential. Biochem. Pharmacol. 79, 77–89. 10.1016/j.bcp.2009.09.014 19765548

[B7] Bush T. G. Puvanachandra N. Horner C. H. Polito A. Ostenfeld T. Svendsen C. N. (1999). Leukocyte Infiltration, Neuronal Degeneration, and Neurite Outgrowth after Ablation of Scar-Forming, Reactive Astrocytes in Adult Transgenic Mice. Neuron 23, 297–308. 10.1016/s0896-6273(00)80781-3 10399936

[B38] Christopherson K. S. Ullian E. M. Stokes C. C. A. Mullowney C. E. Hell J. W. Agah A. (2005). Thrombospondins are Astrocyte-Secreted Proteins that Promote CNS Synaptogenesis. Cell 120, 421–433. 10.1016/j.cell.2004.12.020 15707899

[B8] Cordner Z. A. Khambadkone S. G. Boersma G. J. Song L. Summers T. N. Moran T. H. (2019). Maternal High-Fat Diet Results in Cognitive Impairment and Hippocampal Gene Expression Changes in Rat Offspring. Exp. Neurol. 318, 92–100. 10.1016/j.expneurol.2019.04.018 31051155 PMC6588424

[B9] Dresselhaus E. C. Meffert M. K. (2019). Cellular Specificity of NF-Κb Function in the Nervous System. Front. Immunol. 10, 1043. 10.3389/fimmu.2019.01043 31143184 PMC6520659

[B40] Eroglu C. Barres B. A. (2010). Regulation of synaptic connectivity by glia. Nature 468, 223–231. 10.1038/nature09612 21068831 PMC4431554

[B10] Goetz R. Mohammadi M. (2013). Exploring Mechanisms of FGF Signalling through the Lens of Structural Biology. Nat. Rev. Mol. Cel Biol 14, 166–180. 10.1038/nrm3528 PMC3695728 23403721

[B11] Hales C. N. Barker D. J. P. (1992). Type 2 (Non-insulin-dependent) Diabetes Mellitus: the Thrifty Phenotype Hypothesis. Diabetologia 35, 595–601. 10.1007/BF00400248 1644236

[B12] Hennessy B. T. Smith D. L. Ram P. T. Lu Y. Mills G. B. (2005). Exploiting the PI3K/AKT Pathway for Cancer Drug Discovery. Nat. Rev. Drug Discov. 4, 988–1004. 10.1038/nrd1902 16341064

[B13] Howell J. A. Bidwell G. L. 3rd (2020). Targeting the NF-Κb Pathway for Therapy of Ischemic Stroke. Ther. Deliv. 11, 113–123. 10.4155/tde-2019-0075 31928138

[B14] Jiang Y. Lin L. Liu N. Wang Q. Yuan J. Li Y. (2020). FGF21 Protects against Aggravated Blood-Brain Barrier Disruption after Ischemic Focal Stroke in Diabetic Db/db Male Mice via Cerebrovascular PPARγ Activation. Ijms 21, 824. 10.3390/ijms21030824 PMC7037567 32012810

[B15] Kaltschmidt B. Kaltschmidt C. (2009). NF- B in the Nervous System. Cold Spring Harbor Perspect. Biol. 1, a001271. 10.1101/cshperspect.a001271 PMC2773634 20066105

[B16] Karin M. Ben-Neriah Y. (2000). Phosphorylation Meets Ubiquitination: The Control of NF-Κb Activity. Annu. Rev. Immunol. 18, 621–663. 10.1146/annurev.immunol.18.1.621 10837071

[B17] Karin M. Lawrence T. Nizet V. (2006). Innate Immunity Gone Awry: Linking Microbial Infections to Chronic Inflammation and Cancer. Cell 124, 823–835. 10.1016/j.cell.2006.02.016 16497591

[B18] Katso R. Okkenhaug K. Ahmadi K. White S. Timms J. Waterfield M. D. (2001). Cellular Function of Phosphoinositide 3-Kinases: Implications for Development, Immunity, Homeostasis, and Cancer. Annu. Rev. Cel Dev. Biol. 17, 615–675. 10.1146/annurev.cellbio.17.1.615 11687500

[B39] Koehler R. C. Roman R. J. Harder D. R. (2009). Astrocytes and the Regulation of Cerebral Blood Flow. Trends Neurosci. 32, 160–169. 10.1016/j.tins.2008.11.005 19162338

[B19] Li X. (2019). The FGF Metabolic axis. Front. Med. 13, 511–530. 10.1007/s11684-019-0711-y 31495905 PMC7102389

[B20] Lin C. Shao B. Zhou Y. Niu X. Lin Y. (2016). Maternal High-Fat Diet Influences Stroke Outcome in Adult Rat Offspring. J. Mol. Endocrinol. 56, 101–112. 10.1530/JME-15-0226 26643911

[B21] Lin C. Wu X. Zhou Y. Shao B. Niu X. Zhang W. (2018). Maternal High-Fat Diet Programs Cerebrovascular Remodeling in Adult Rat Offspring. J. Cereb. Blood Flow Metab. 38, 1954–1967. 10.1177/0271678X17731956 28914129 PMC6259319

[B22] Myer D. J. Gurkoff G. G. Lee S. M. Hovda D. A. Sofroniew M. V. (2006). Essential Protective Roles of Reactive Astrocytes in Traumatic Brain Injury. Brain 129, 2761–2772. 10.1093/brain/awl165 16825202

[B23] Ng S.-F. Lin R. C. Y. Laybutt D. R. Barres R. Owens J. A. Morris M. J. (2010). Chronic High-Fat Diet in Fathers Programs β-cell Dysfunction in Female Rat Offspring. Nature 467, 963–966. 10.1038/nature09491 20962845

[B24] Omran A. R. (1971). The Epidemiologic Transition: A Theory of the Epidemiology of Population Change. Milbank Memorial Fund Q. 49, 509–538. 10.2307/3349375 5155251

[B25] Pekny M. Pekna M. (2016). Reactive Gliosis in the Pathogenesis of CNS Diseases. Biochim. Biophys. Acta (Bba) - Mol. Basis Dis. 1862, 483–491. 10.1016/j.bbadis.2015.11.014 26655603

[B26] Pekny M. Wilhelmsson U. Tatlisumak T. Pekna M. (2019). Astrocyte Activation and Reactive Gliosis-A New Target in Stroke. Neurosci. Lett. 689, 45–55. 10.1016/j.neulet.2018.07.021 30025833

[B27] Prabhakaran S. Ruff I. Bernstein R. A. (2015). Acute Stroke Intervention. JAMA 313, 1451–1462. 10.1001/jama.2015.3058 25871671

[B28] Rabinstein A. A. (2017). Treatment of Acute Ischemic Stroke. CONTINUUM: Lifelong Learn. Neurol. 23, 62–81. 10.1212/CON.0000000000000420 28157744

[B29] Sacks D. Sacks D. Baxter B. Campbell B. C. V. Carpenter J. S. Cognard C. (2018). Multisociety Consensus Quality Improvement Revised Consensus Statement for Endovascular Therapy of Acute Ischemic Stroke. AJNR Am. J. Neuroradiol 39, E61–E632. 10.1177/174749301877871310.3174/ajnr.A5638 29773566 PMC7410632

[B30] Sofroniew M. V. (2009). Molecular Dissection of Reactive Astrogliosis and Glial Scar Formation. Trends Neurosciences 32, 638–647. 10.1016/j.tins.2009.08.002 PMC2787735 19782411

[B31] Staiger H. Keuper M. Berti L. Hrabě de Angelis M. Häring H.-U. (2017). Fibroblast Growth Factor 21-Metabolic Role in Mice and Men. Endocr. Rev. 38, 468–488. 10.1210/er.2017-00016 28938407

[B32] Suzuki K. (2018). The Developing World of DOHaD. J. Dev. Orig Health Dis. 9, 266–269. 10.1017/S2040174417000691 28870276

[B37] Ullian E. M. Sapperstein S. K. Christopherson K. S. Barres B. A. (2001). Control of Synapse Number by Glia. Science 291, 657–661. 10.1126/science.291.5504.657 11158678

[B33] Wang D. Liu F. Zhu L. Lin P. Han F. Wang X. (2020). FGF21 Alleviates Neuroinflammation Following Ischemic Stroke by Modulating the Temporal and Spatial Dynamics of Microglia/macrophages. J. Neuroinflammation 17, 257. 10.1186/s12974-020-01921-2 32867781 PMC7457364

[B34] Wang H. Xiao Y. Fu L. Zhao H. Zhang Y. Wan X. (2010). High-level Expression and Purification of Soluble Recombinant FGF21 Protein by SUMO Fusion in *Escherichia coli* . BMC Biotechnol. 10, 14. 10.1186/1472-6750-10-14 20163718 PMC2831817

[B35] Wang Q. Yuan J. Yu Z. Lin L. Jiang Y. Cao Z. (2018). FGF21 Attenuates High-Fat Diet-Induced Cognitive Impairment via Metabolic Regulation and Anti-inflammation of Obese Mice. Mol. Neurobiol. 55, 4702–4717. 10.1007/s12035-017-0663-7 28712011 PMC5971086

[B36] Yu Y. Bai F. Wang W. Liu Y. Yuan Q. Qu S. (2015). Fibroblast Growth Factor 21 Protects Mouse Brain against D-Galactose Induced Aging via Suppression of Oxidative Stress Response and Advanced Glycation End Products Formation. Pharmacol. Biochem. Behav. 133, 122–131. 10.1016/j.pbb.2015.03.020 25871519

[B42] Zhou B. Zuo Y-X. Jiang R-T. (2019). Astrocyte Morphology: Diversity, Plasticity, and Role in Neurological Diseases. CNS Neurosci. Ther. 25, 665–673. 10.1111/cns.131 30929313 PMC6515705

